# Vitamin E Content and Composition in Tomato Fruits: Beneficial Roles and Bio-Fortification

**DOI:** 10.3390/ijms161226163

**Published:** 2015-12-08

**Authors:** Assunta Raiola, Gian Carlo Tenore, Amalia Barone, Luigi Frusciante, Maria Manuela Rigano

**Affiliations:** 1 Department of Agricultural Sciences, University of Naples Federico II, Via Università 100, Portici (Naples) 80055, Italy; assuntaraiola@hotmail.com (A.R.); ambarone@unina.it (A.B.); fruscian@unina.it (L.F.); 2 Department of Pharmacy, University of Naples Federico II, Via D. Montesano 49, Naples 80131, Italy; giancarlo.tenore@unina.it

**Keywords:** tocopherol, tocotrienol, *Solanum lycopersicum*, health effects, metabolic pathway, biosynthesis, environmental conditions, processing, biofortification, quantitative trait loci

## Abstract

Several epidemiological studies have demonstrated that high vitamin E intakes are related to a reduced risk of non-communicable diseases, while other dietary antioxidants are not, suggesting that vitamin E exerts specific healthy functions in addition to its antioxidant role. In this regard, tomato (*Solanum lycopersicum*), one of the most consumed vegetables of the whole world population, is an important source of both tocopherols and tocotrienols. However, vitamin E content may strongly depend on several biotic and abiotic factors. In this review we will debate the elements affecting the synthesis of tocopherols and tocotrienols in tomato fruit, such as environmental conditions, genotype, fruit maturity level, and the impact of classical processing methods, such as pasteurization and lyophilization on the amount of these compounds. In addition we will analyze the specific vitamin E mechanisms of action in humans and the consequent functional effects derived from its dietary intake. Finally, we will examine the currently available molecular techniques used to increase the content of vitamin E in tomato fruit, starting from the identification of genetic determinants and quantitative trait loci that control the accumulation of these metabolites.

## 1. Introduction

The FAO (Food and Agriculture Organization of the United Nations) and the WHO (World Health Organization) have highlighted the function of nutrition and, in particular, of plant-derived phytochemicals in the prevention of non-communicable diseases and cancer [1]. In this regard, tomato (*Solanum lycopersicum*), one of the most globally-consumed fruits, is an important source of compounds with recognized healthy effects, such as reduced risk of cardiovascular diseases, digestive tract tumors, inflammatory processes, cardiovascular diseases, hypertension, diabetes, and obesity [2]. These properties have been associated to the presence of hydrophilic (mainly ascorbic acid and phenols) and lipophilic antioxidants, including carotenoids (mainly lycopene and β-carotene) and vitamin E (VTE) [3].

Structural analyses have revealed that VTE consists of four tocopherols (α, β, δ, and γ), and four tocotrienols (α, β, δ, and γ) that are non-enzymatic lipid-soluble antioxidants. In both food and human tissues, α-tocopherol and γ-tocopherol are the more abundant VTE forms. Interestingly, the α-tocopherol shows the highest biological activity compared to the other VTE forms. This is due to the selective retention of this compound mediated by the hepatic α-tocopherol transfer protein (α-TTP) that is considered the main VTE regulator in humans, while the other VTE forms are degraded and excreted by the liver. The selective incorporation of α-tocopherol into circulating lipoproteins, that distribute this compound to non-hepatic tissues, is favored by hepatic α-TTP [4,5].

All four forms of tocopherols consist of a polar chromanol head group placed at the membrane surface and a hydrophobic phytyl tail situated in the membrane core, and diverge only in the positions, number and methyl groups at the chromanol ring [6]. Tochoperols are synthesized only by photosynthetic organism and are able to inhibit lipid peroxidation, to contribute to membrane stability, fluidity, and permeability and to protect the photosystem II from oxidative damage by scavenging lipid peroxyl radicals and singlet oxygen [7,8]. In plants, these antioxidants play a role in different physiological phenomena such as plant growth, development, and senescence [9]. A high variability has been reported about the content of tocochromanol and composition in plant tissues [10]. In particular, photosynthetic tissues generally show low levels of total tocochromanols (<50 μg/g fresh weight) and a high percentage of α-tocopherol, while seeds contain 10–20 times more of total tocochromanols [11].

Epidemiological studies have reported that high VTE intakes are linked to a reduced risk of cardiovascular diseases, whereas intakes of other dietary antioxidants (such as vitamin C and β-carotene) are not, suggesting that VTE exerts specific functions in addition to its antioxidant role [12]. The Institute of Medicine report on VTE [13] has reported a Recommended Dietary Allowance (RDA) of around 15 mg/day of α-tocopherol. Interestingly, it has been observed that the advantageous properties associated with the consumption of tomatoes are related to the synergistic properties of the tomato molecules, in particular lycopene and α-tocopherol, which have been shown to inhibit HL-60 (human promyelocytic leukemia cells) cell differentiation, low-density lipoprotein (LDL) oxidation, and prostate carcinoma cell proliferation [14].

As already known, antioxidants amount in tomato may depend on many biotic and abiotic factors, including environmental conditions [15], genotype [16], fruit maturity level [17], and cultivation practice [18]. In this review we will focus our attention on several aspects concerning tocopherols in tomato. In particular, we will debate the factors influencing the synthesis of these molecules in tomato fruit, their beneficial effects and the impact of common processing methods on their content. In addition, we will examine the currently available molecular techniques adopted to enhance the content of VTE in tomato fruits.

## 2. Beneficial Roles of Vitamin E

Several recent human studies have highlighted the potential beneficial role of VTE intake on inflammation-related diseases and cardiac functionality as summarized in Table 1.

These effects are mainly due to VTE antioxidant ability since it inhibits the production of ROS (reactive oxygen species) molecules when fat undergoes oxidation and during the propagation of free radical reactions [19]. In particular VTE is located in the cell and organelle membranes where it can exert its maximum protective effect, acting as the first line of defence against lipid peroxidation [20]. Howard *et al.* [20] demonstrated that VTE promotes membrane repair by preventing the formation of oxidised phospholipids that might interfere with the membrane fusion events. Another study [21] reported that adenosine diphosphate-induced platelet aggregation significantly decreased in healthy people who received VTE enriched in γ-tocopherol. Other evidence showed that VTE could decrease the risk of type-2 diabetes and prostate cancer [3,22,23]. Important effects are related to the stimulation of body’s defenses and the enhancement of humoral and cell immune responses [19]. Conversely, VTE deficiency can cause anemia due to the oxidative damage to the red blood cells, impairment of the immune response, retinopathy, and cataracts [24]. Interestingly, the presence of VTE in plasma is associated with a reduced risk of Alzheimer’s disease in older patients [19].

The tocopherol transfer protein (TTP) is a critical regulator of VTE status stimulating its movement between membrane vescicles *in vitro* and regulating the secretion of tocopherol from hepatocytes. Indeed, heritable mutations in the *ttpA* gene (encoding α-TTP) cause ataxia with VTE deficiency (AVED), an autosomal recessive disease characterized by low plasma VTE levels and progressive neurodegeneration [25]. In these cases, after a regular consumption of VTE supplements, the concentration in plasma normalizes in few hours. Usually, a daily α-tocopherol dose of 800–1200 mg is sufficient to prevent further reduction of neurologic function [4]. The advantage of VTE supplements in individuals who are not affected by deficiency is controversial. For example, Miller *et al*. [26] reported that high-dosage (>400 International Unit (IU)/day, where 1 IU of α-tocopherol is equivalent to 0.67 mg of the natural form [27]) VTE supplements may increase all-cause mortality in adults with chronic diseases and should be avoided, even though precise estimation of the threshold at which risk increases is difficult. In any case these findings may not be generalizable to healthy adults.

The significant correlation between supplementation with VTE alone and a reduced incidence of myocardial infarction, fatal myocardial infarction, and non-fatal myocardial infarction in humans has been evidenced in a recent meta-analysis conducted by Loffredo *et al.* [28]. The meta-analysis was performed comparing a sample supplemented with VTE alone (group A) with one supplemented with VTE plus other antioxidant agents such as multivitamins, vitamin A, vitamin C, β-carotene, *n*-3 polyunsaturated fatty acids and ramipril and selenium (group B). Compared with control, group A showed a significant reduction of myocardial infarction (3.0% *vs.* 3.4%) and was associated with an absolute risk reduction (ARR) of 0.36%. No significant reduction was demonstrated comparing subjects treated with a combination of antioxidants (group B) *versus* controls. A significant reduction of myocardial infarction was observed in group A only for high VTE dosage (≥400 IU/day).

A study of Magosso *et al.* [29] has demonstrated the efficacy of mixed tocotrienols in normalizing the hepatic echogenic response in hypercholesterolemic patients affected by nonalcoholic fatty liver disease (NAFLD). Eighty-seven untreated hypercholesterolemic adults with ultrasound-proven NAFLD were enrolled into control group (*n* = 44) and tocotrienols group (*n* = 43) for one year. The present study showed that the patients that received 200 mg of tocotrienols twice daily, have significantly benefited in terms of normalization of the hepatic echogenic response in NAFLD compared to placebo.

Another recent meta-analysis has indicated VTE supplementation as a potentially good strategy for decreasing inflammatory conditions in susceptible people [30]. Particularly, the results of this meta-analysis suggest that supplementation with VTE in the form of either α-tocopherol or γ-tocopherol would reduce the plasma levels of C-reactive protein (CRP), a marker of chronic inflammation, with a major role in the etiology of chronic disease. The effect size of VTE supplementation on CRP level *vs.* control was calculated at −0.62 mg/L. There was a greater reduction in the serum CRP level following supplementation with α-tocopherol (−0.73 mg/L) compared with γ-tocopherol (−0.62 mg/L).

Upritchard *et al.* [31] compared the impact of short-term dietary supplementation with tomato juice and VTE on the susceptibility of LDL to oxidation and levels of CRP and cell adhesion molecules in patients with type-2 diabetes. This study found that the consumption of commercial tomato juice increases the intrinsic resistance of LDL to oxidation almost as effectively as supplementation with a high dose of vitamin E, which also decreases plasma levels of CRP and the risk of myocardial infarction in patients.

It is worth saying that the intake of tocopherols from tomato matrix can exert a different impact on health compared to the single VTE supplementation, due to the co-occurrence of other antioxidant molecules in tomato fruit. Indeed, Zanfini *et al.* [14] studied the antioxidant activity of lipophilic extracts from different tomato varieties and reported a high synergistic effect of the α-tocopherol-lycopene mixtures. The authors speculate that this effect could be due to the fact that electrons can transfer from the carotenoid to the α-tocopherolxyl radical to regenerate α-tocopherol.

A similar mechanism has been suggested for the interactions between vitamin C, carotenoids, and α-tocopherol [32]. A synergistic interaction between ascorbic acid and α-tocopherol was also observed during the process of lipid peroxidation [33] and during the inhibition of the inflammation process [34].

Altogether, these studies demonstrate the importance of an appropriate VTE intake by humans strengthening the knowledge of the role of dietary antioxidants in maintaining better general health.

**Table 1:** Main mechanisms of action and functional roles of vitamin E.

Compounds	Effect	Reference
VTE group	Enhanced humoral and cell immune responses reduced risk of Alzheimer’s disease	[19]
	Membrane repair by preventing the formation of oxidised phospholipids	[20]
	Reduced risks of type 2 diabetes and prostate cancer	[3,22,23]
	Prevention of retinopathy and cataracts	[24]
	Reduced risk of myocardial infarction	[28]
α-Tocopherol	Inhibition of HL-60 leukemic cell differentiation, reduced risk of low-density lipoprotein (LDL) oxidation and prostate carcinoma cell proliferation	[14]
	Reduction in the serum C-reactive protein level	[30]
γ-Tocopherol	Decreased adenosine diphosphate-induced platelet aggregation	[21]
Mixed tocotrienols	Normalized hepatic echogenic response	[29]

## 3. Factors Affecting Vitamin E Content in Tomato Fresh Fruit

The European Food Safety Authority (EFSA) has finalized its Dietary Reference Values (DRVs) for VTE (α-tocopherol) and recommends intakes of 13 mg α-tocopherol/day for men and 11 mg/day for women. These values are also recommended for children older than 10 years. These values are not easily achieved with the common diet. Indeed, in European countries α-tocopherol intake in adults is estimated between 7.8 and 12.5 mg/day in women and between 8.2 and 16 mg/day in men [35]. Three national surveys and the Continuing Survey of Food Intakes by Individuals have found that the diets of the majority of people residing in USA provide levels of vitamin E lower than RDA (recommended dietary allowance) levels [27]. Therefore, it is of great interest to examine the factors that can affect VTE amount in largely consumed plant foods, such as tomato. Several studies have reported that in unpeeled tomato fruit the mean VTE content was around 60 mg/kg dry weight (DW) or 3 mg/kg fresh weight (FW) with 95% confidence limits of 35–100 mg/kg [36]. The tocopherol content in whole tomato fruit was found to be moderately high, *i.e.*, about 0.86 mg/100 g FW [37]. Frusciante *et al.* [38] reported a VTE level in tomatoes between 0.17 and 0.62 mg/100 g FW. However, tocopherol content and composition differ greatly in different plant tissues. Generally, the two major tocopherols found in plant tissues are α- and γ-tocopherols. In green leaves, mainly α-tocopherol is found, while γ-tocopherol is the most common tocopherol in seeds [39]. Quadrana *et al.* [7] found that α-tocopherol was the most abundant form in leaves and fruits contributing up to 97% of the total VTE in the mature green fruits. Pék *et al.* [40] found four components of tocochromanol in cherry tomato samples including α-tocopherol, γ-tocopherol, β-tocopherol, and γ-tocotrienol. α-Tocopherol represented 48%–70% of total tocopherols. The proportion of γ-tocopherol was between 22% and 38%, while the remaining two were lower than 10%. Rain-fed control plants showed higher α-tocopherol concentration compared to regularly irrigated plants, possibly through the dilution effect of enlarged fruits. On the contrary irrigation increased γ-tocopherol ratio of tocopherol concentration.

The level of tocopherols is strongly linked to plant physiological state, stress intensity and species-specific sensitivity to stress [6] (Table 2). Environmental stress such as drought, high light intensity, low temperature, salinity, and heavy metal ions may enhance the content of ROS. In fact, a brief or prolonged increase in steady-state level of ROS, called oxidative stress, causes alterations of cellular signaling core metabolic processes determining oxidation of cellular constituents [41]. For example, high light intensity stress excessively excites chlorophylls generating ROS, which may attack various cellular components such as polyunsaturated fatty acids (PUFA) [42]. Tocopherols protect them against photo-oxidation by quenching and scavenging ROS and alkyl peroxy radicals generated during photosynthesis [43,44]. The protective effects of VTE include not only singlet oxygen scavenging but also a general protection of thylakoid membranes by tocopherol molecules in the lipid phase [45]. Loyola *et al.* [44] demonstrated that α-tocopherol biosynthesis is part of the adaptation mechanisms to drought stress of *Solanum chilense*, a wild tomato genotype. Skłodowska *et al.* [45] studied the function of chloroplastic tocopherol in the response of tomato plants to different degrees of NaCl stress. Starting from the second day in the salt stressed plants a higher tocopherol level was observed. Under moderate salinity stress, an increase in its content was found on the second day (193% of the control), while under severe stress greatest changes were reported on the fifth and seventh days (from 438% to 355% of the control value), respectively.

Del Giudice *et al.* [46] reported that the content of antioxidant compounds in tomatoes is strongly influenced by on-vine ripening. More specifically, Dumas *et al.* [17] found that the ripening stage had an important impact on the level of various forms of tocopherols in tomato. In particular, the α-tocopherol content increased from 1.5 (green stage) to 3.2 mg/kg FW (red stage), while the β-tocopherol content increased from nearly 0 (green stage) to 0.3 mg/kg FW (red stage); the γ-tocopherol content amount from 0.7 (green stage) to 1.4 mg/kg (yellow stage) before decreasing to 1 mg/kg (red stage) [17]. Quadrana *et al.* [7] found that α-tocopherol level and total tocopherol did not vary across development, while β-tocopherol decreased from green to mature green stages and δ- and γ-tocopherol increased upon mature green stage ten-fold and five-fold, respectively. The authors explained these observed changes as due to the reduction of γ-tocopherol methyltransferase mRNA and the up-regulation of the gene coding for the enzyme 2-methyl-6-phytyl-1,4-benzoquinol methyltransferase.

Caretto *et al.* [47] studied the effect of potassium (*K*) and cultivar on some quality parameters of tomato including VTE. In this experiment, three cultivars (SVR, Kabiria, and Esperanza) growing in a soilless system at three *K* levels were compared. Kabiria showed a total tocopherol content (18.5 mg/kg) significantly higher than SVR and Esperanza cultivars (12.2 and 10.3 mg/kg, respectively). An increase in the *K* level in the nutrient solution from 300 to 450 mg/L seemed to inhibit the synthesis and/or accumulation of total tocopherols in all three tomato varieties tested. As previously reported by Seybold *et al.* [39], the isoforms α and γ resulted in more than 94% of the total tocopherol content of tomato fruits and γ-tocopherol content was not far from the α-levels, since γ-tocopherol is extensively present in seeds. However, there is very little information in the literature on the effects of different fertilizers on the antioxidant content in tomatoes.

Amount of tocopherols differs significantly according to varieties of tomato examined. Lenucci *et al.* [48] analyzed fourteen cultivars of cherry tomatoes and four cultivars of high-pigment hybrids. The highest values (10 mg/kg FW; 113 mg/kg DW) of VTE was observed in cultivar LS203, where α-tocopherol content appeared to be exceptionally higher than that observed in the other cherry cultivars tested, while the lowest values of α-tocopherol were estimated in Salentino (2 mg/kg FW, 25 mg/kg DW) and Sharon (2 mg/kg FW, 29 mg/kg DW) cultivars. A high content of α-tocopherol was also detected in high pigment tomato hybrids containing high levels of lycopene [48]. Maršić *et al.* [49] detected three tocopherols (α, δ, and γ-tocopherol) in several varieties of tomato fruits grown under different climatic conditions. Significant differences in the average content of total tocopherols were detected for the varieties Sun, Sunjay, Chaser, Empire, and San Marzano. The data revealed that oval and elongated fruit grown in areas with higher amounts of solar radiation, higher average air temperature, and low precipitation amount during the ripening period correlated positively with VTE content.

**Table 2:** Main factors influencing VTE content.

Factor	Effect	Reference
Ripening	Increasing of α and β-tocopherols from green to red stage; increasing of γ-tocopherol from green to yellow stage	[17]
Water	Increasing of α-tocopherols in rain fed plants; increasing of γ-tocopherol after irrigation	[40]
Light	Increasing of tocopherols under high light intensity	[41]
Temperature	Increasing of tocopherols under low temperature	
salt (NaCl)	Increasing of tocopherols under salinity stress	[45]
Potassium	Inhibition of synthesis and/or accumulation of total tocopherols under high potassium level	[47]
Variety	Different level α-tocopherol in different tomato cultivars	[48,49]

Today, it seems difficult to define optimum growth conditions to optimize the biosynthesis and storage of VTE in the tomato fruit. More data to clearly understand how agronomic factors and techniques may interfere with the effects of light, temperature, and genetic background are needed, since all these factors combined are responsible for VTE accumulation in the fruit.

## 4. Impact of Processing

In the last few years, tomato consumption has increased since tomato fruits supply both the fresh market and processing products, such as sauces, juices, purees. The Economic Research Service of the USDA estimates that 35% of raw tomatoes are processed into sauces, 18% into tomato paste, 17% into canned tomatoes and 30% into juices and ketchup [2]. Several studies reported that some bioactive compounds of tomato fruits, such as carotenoids, are more abundant after thermal processing, since heat treatment and/or homogenization can disrupt the cellular matrix of tomato fruit changing the bioavailability of different nutrients [50,51].

Abushita *et al.* [18] found a loss of α-tocopherol of 20.3% during thermal processing of tomato paste, while a loss of 46.5%of α-tocopherol quinone and of 32.7% of γ-tocopherol were recorded. Capanoglu *et al.* [52] reported that α-tocopherol was not affected by the industrial processing, while its biosynthetic precursor γ-tocopherol was significantly lower in paste than in fresh tomato fruit. In addition, the authors found that the concentration of γ-tocopherol was relatively high in the seed and skin fraction while amount of α-tocopherol was similar in all the fractions. For this reason γ-tocopherol can easily be lost during processing steps, which include the removal of the seeds. Boiling can increase the amount of total tocopherols from 0.68 to 0.76 mg/100 g edible weight, up to 4.98 mg/100 g in canned pastes according to a study carried out in United States. However, fruits of processing varieties are generally characterized by higher levels of α-tocopherol than the salad tomatoes [53]. Homogenization and sterilization of tomatoes during production of tomato juice have been reported to be characterized by significant losses of α-tocopherol content. However, short-term heating of tomato soup, tomato sauce, and baked tomato slices from Spanish tomatoes led to a significant rise of α-tocopherol level during the cooking of the sauce [39]. Possibly, α-tocopherol was released from its binding sites as the effect of thermal treatment, and only heating for >1 h or at oven temperatures >200 °C causes a thermal degradation. In addition, the α-tocopherol contents decreased during production of tomato juice. This result may be due to the fact that the juice was produced from preheated tomatoes and so the complete release of α-tocopherol by thermal processing had already been reached. Tocopherol stability was also investigated in dried tomato pulp and peel stored at 30 °C with various water activity (aw) levels [54]. After 39 days of storage at aw = 0.17 the retention of α-tocopherol was 60% and 26% in the pulp and in peel, respectively, and the stability of α-tocopherol increased with increasing aw. Hwang *et al.* [55] investigated the influence of thermal processing on the assessment of tocopherols at 100, 130, and 160 °C for five, 10, and 20 min, then freeze-dried. The mean value of α-tocopherol, and γ-tocopherol in fresh tomatoes were 21.2, 1.1, 2.7, 8.0, and 2.5 mg/100 g dry weight, respectively. Oven baking of tomato at 160 °C for 20 min caused a significant increase in the level of α-tocopherol level by 32%. These data suggested that thermal processes might break down cell walls improving the release of tocopherols from tomato fruit. In addition, lyophilization of samples may increase the extractability of tocopherols. These molecules have been also found in processing wastes. On a dried weight basis, tomato wastes contained increased amount of tocopherols, compared to whole tomatoes. In particular α-tocopherol, (β + γ)-tocopherol and δ-tocopherol showed mean levels of 155.07, 22.0, 0.23 mg/kg DW, respectively, in wastes, while in whole tomatoes the mean amount were 85.8, 6.7, and 0.12 mg/kg DW for the same compounds. Therefore, these constituents could be isolated from tomato wastes and then utilized as natural antioxidants for the formulation of functional foods [56].

## 5. Biosynthetic Pathways of Vitamin E

The tocochromanol biosynthetic pathway was clarified from radio tracer studies carried out in chloroplasts and cyanobacteria in the1980s [10,57]. These compounds are synthesized, in whole or in part, from the plastidic isoprenoid biosynthetic pathway. In fact, in plants, tocochromanol and also the enzymes of the core pathway, have been found only in plastids. Since it has not yet been demonstrated that any isoform can be carried within the plant [58], it is accepted that the biosynthesis also occurs in this district. VTE compounds contain a polar chromanol group originated from homogentisate, which derives from shikimate pathway, and a polyprenyl lipophilic side chain which results from the plastidial methylerythritol 4-phosphate (MEP) pathway [7,58,59]. Tochopherols have saturated tails derived from phytyl-PP (phytyl pyrophosphate), whereas tocotrienols have an unsaturated tail derived from geranylgeranyl 2P.

**Figure 1:**
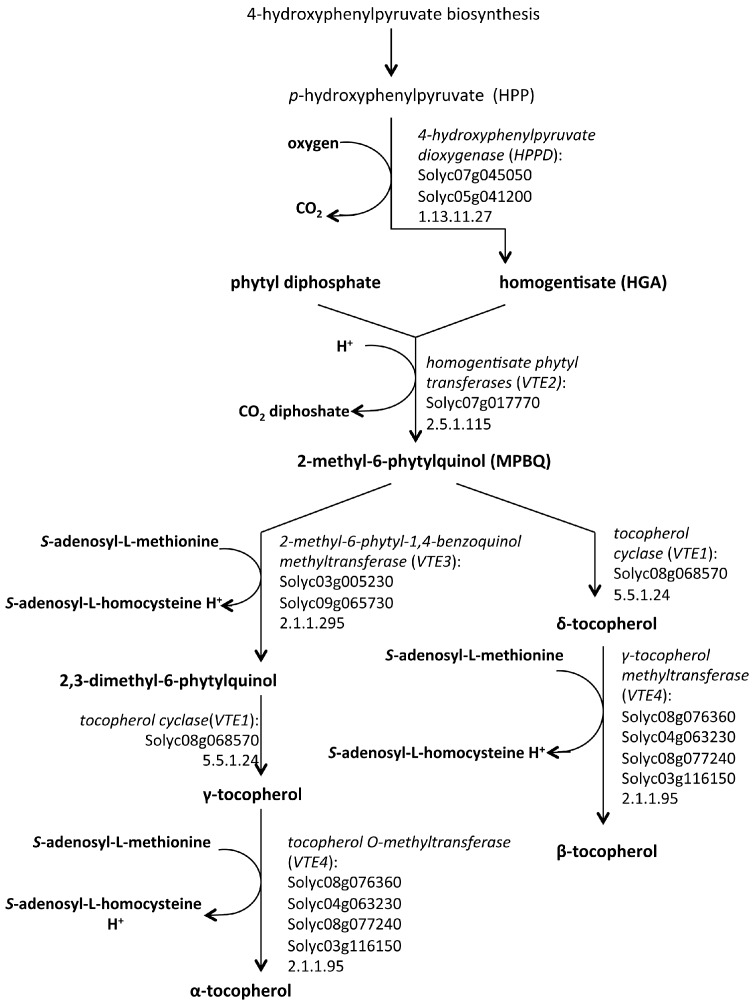
The tocopherol biosynthetic pathway in *Solanum lycopersicum* as described in MetaCyc [61].

**Figure 2:**
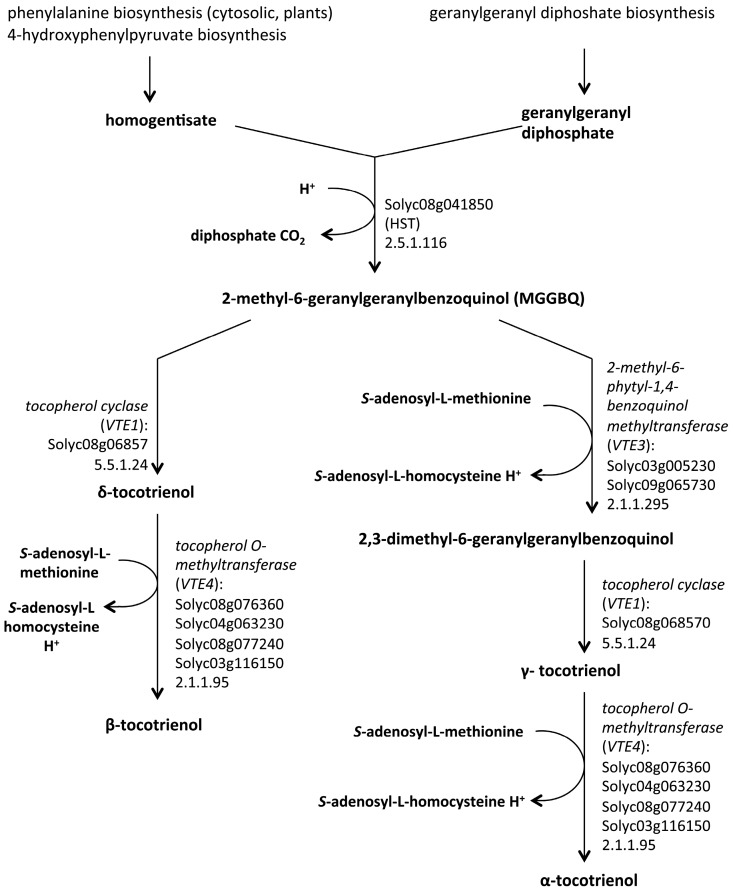
The tocotrienol biosynthetic pathway in *Solanum lycopersicum* as described in MetaCyc [61].

For the headgroup synthesis, *p*-hydroxyphenylpyruvate (HPP) is converted into homogentisate (HGA) by the enzyme HPP dioxygenase (HPPD). Successively, phytyl-PP or GGDP are condensed with HGA by homogentisate phytyl transferases (VTE2) to produce 2-methyl-6-phytylquinol (MPBQ) and 2-methyl-6-geranylgeranylbenzooquinol (MGGBQ) that are intermediates in tocopherol and tocotrienol synthesis, respectively. The VTE biosynthesis pathway from the reduction of hydroxyphenylpyruvate to homogentisate is known as the “VTE core pathway”. The four forms of tocopherol are products of the reactions catalysed by 2-methyl-6-phytyl-1,4-benzoquinol methyltransferase (VTE3), tocopherol cyclase (VTE1) and γ-tocopherol methyltransferase (VTE4) that converts δ- and γ-tocopherols (and tocotrienols) to β- and α-tocopherols (and tocotrienols), respectively [7,60,61] (Figure 1 and Figure 2).

In the last decade, the genes encoding for the enzymes of the majority of the steps in the VTE pathway have been identified and cloned by the use of genetic and genomics-based methods. However, most of this knowledge is relative to the model organisms *Arabidopsis thaliana* and *Synechocystis* sp. PCC6803 [62]. In tomato, Almeida *et al.* [63] identified, characterized, and mapped the enzyme encoding the genes involved in VTE biosynthetic pathways.

Subsequently, Quadrana *et al.* [7] by using a dedicated qPCR array platform determined the transcript levels of 47 genes involved in tocopherol biosynthesis in tomato leaves and across fruit development. Results were analyzed in parallel with tocopherol and pigment profiles. The authors highlighted the key role of 18 key genes from MEP (methylerythritol phosphate), SK(shikimate), phytol recycling, and VTE-core pathway. In particular, it was demonstrated the key role of the genes coding for the 1-deoxy-d-xylulose-5-phosphate synthase and for the geranylgeranyl reductase that determine phytyl-diphosphate availability together with enzyme encoding genes involved in chlorophyll-derived phytol metabolism [7]. In another study, Lu *et al.* [64] demonstrated that the homogentisate phytyl-transferase is a limiting enzymatic step in the VTE pathway, using metabolic engineering by stable transformation of the tomato chloroplast genome. The same authors demonstrated that in transplastomic plants accumulating higher VTE levels there was also an increase in the levels of carotenoids and chlorophylls. Several studies have revealed a strict connection between VTE and the pathways of photosynthetic pigment biosynthesis. Isoprenoids are precursors in chlorophyll, carotenoid, and tocopherol pathways; therefore, tocopherol content can be deeply affected by changes in carotenoid biosynthesis and chlorophyll metabolism. For example, Fraser *et al.* [65] demonstrated that tomato fruits overexpressing phytoene synthase (PSY), a key enzyme in carotenoid biosynthesis, showed increased levels of tocopherols. Almeida *et al.* [63] explored the metabolic network that links isoprenoid biosynthesis and recycling in ripening impaired, senescence-related, and jasmonate-insensitive tomato mutants and demonstrated that fruits from the tomato mutants have altered tocopherol content and composition.

## 6. Biofortification of Vitamin E Content

Due to the high nutritional value and the benefits of VTE in human health, obtaining novel tomato plant lines that accumulate higher levels of VTE in the fruits has long been the focus of conventional breeding programs and metabolic engineering approaches [3,64].

Today a vast array of genetic resources are available for tomato, these includes populations of genetically defined genotypes generated through crossing *S. lycopersicum* with wild relatives, mutant collections and diverse range of mutants generated using TILLING (targeting induced local lesions in genomes) platforms [66]. Biofortified tomato plants can be obtained by taking advantage of the natural variation of plant genomes. Tomato nutritional quality usually exhibits quantitative variation controlled by several genes involved in many of the primary and secondary metabolism pathways and regulated by developmental, physiological and environmental signals. Therefore, one main focus of several genetic studies has been the identification of genetic determinants and quantitative trait loci (QTL) that control the accumulation in tomato fruit of VTE [3,7]. Population of introgression lines (ILs) derived from wild tomato species (such as *S. pimpinellifolium*, *S. habrochaites*, and *S. pennellii*) have been used as a tool to dissect quantitative traits in their main genetic components, thus allowing the identification of those mostly influencing the studied traits [67,68]. A set of nearly isogenic lines were previously constructed in which marker-defined chromosome segments of the wild species *Solanum pennellii* were replaced with homologous intervals of the cultivated variety M82 [67]. The *S. pennellii* ILs were used to map several QTLs associated with valuable traits that could be introduced into modern varieties to improve specific traits characters related to tomato fruit quality [69,70]. Schauer *et al.* [71] reported a detailed metabolite profile of 76 *Solanum pennellii* introgression lines and identified two QTLs explaining variation in α-tocopherol fruit content located on chromosomes 6 and 9. Successively, Almeida *et al.* [63] studied variations of tocopherol isoforms (α, β, γ, and δ) in ripe fruits of the same introgression lines. The authors confirmed the VTE QTL on chromosomes 6 and 9 and identified novel QTL on chromosomes 7 and 8. In addition, Perez-Fons *et al.* [66] described a multi-platform metabolomics analysis of ILs, using NMR (nuclear magnetic resonance), mass spectrometry, and HPLC, (high performance liquid chromatography) and quantified alleles responsible for tochopherol content in the fruit. In this way, the authors identified two ILs (IL 8-2 and IL 8-2-1) exhibiting high levels of γ-tocopherol in the fruit, probably due to the presence of a wild γ-methyl tocopherol transferase gene located in the introgressed wild region. Recently, Quadrana *et al.* [7] reported the fine mapping of the metabolic QTL located on to the introgressed region of the *S. pennellii* IL9-2-6 (mQTL9-2-6) to a locus encoding VTE3, a gene that encodes a 2-methyl-6-phytylquinol methyl transferase which is a central enzyme for α-tocopherol and γ-tocopherol synthesis. The authors demonstrated that mQTL9-2-6 is a QTL associated with differential methylation of a SINE retrotransposon that is located in the promoter region of the alleles VTE3(1). DNA methylation of the promoter can be spontaneously reverted, and this can lead to different epialleles affecting gene expression and VTE content in tomato fruits. This work highlighted the importance of epigenetics in determining agronomic traits and demonstrated that naturally-occurring epialleles are responsible for regulation of a nutritionally-important metabolic QTL [7]. Despite the conventional breeding is a good strategy to improve the quality of tomato fruit, this techniques has some limitations due to sexual incompatibility and needs long breeding programs, whereas genetic engineering has no such drawbacks and new genes can be introduced directly into local varieties. Plant metabolic engineering can employ different approaches to improve the amount of various phytonutrients in tomato fruits. One strategy to increase the amount of tocopherol in tomato plants was based on the overexpression of biosynthetic genes in transgenic plants. Seo *et al.* [72] overexpressed in transgenic tomato plants a gene coding for HPT (homogentisate phytyltransferase), an important enzyme in the biosynthesis of tocopherols. The authors overexpressed one HPT homolog (MdHPT1) that was previously isolated from apple (*Malus domestica* Borkh. Cv. Fuji). The ectopic expression of MdHPT1 in transgenic tomatoes plants resulted in an increase of the level of α-tocopherol of up to 3.6-fold and 1.7-fold in transgenic leaves and fruits respectively, relative to control tissues.

Another approach in metabolic engineering is the concerted expression of multiple genes of a complex biosynthetic pathway. Chloroplast genes are organized in operons that are co-expressed as polycistronic transcripts and often processed as monocistronic mRNAs. Therefore, in transgenic plastids it is possible to perform efficient metabolic pathway engineering with synthetic multigene operons. Lu *et al.* [64] used stable transformation of the chloroplast genome in tomato to engineer the tochopherol metabolic pathway. The authors overexpressed three key plastid-localized enzymes (homogentisate phytyltransferase, tocopherol cyclase and γ-tochopherol methyltransferase) using single constructs and synthetic operons in tobacco and tomato plants and achieved up to ten-fold increase in total tocochromanol accumulation. Compared to nuclear transformation, chloroplast transformation offers significant advantages, such as high and stable production levels of recombinant proteins, biological containment of transgenes and recombinant products, no gene silencing, and position effects and cellular compartimentalization of compounds harmful to the plant [73].

## 7. Conclusions

Several human studies highlight the beneficial role of vitamin E supporting its anti-inflammatory and anticancer action, whose molecular mechanisms have been abundantly examined and described [19]. A synergistic activity of the tomato phytochemicals has also been demonstrated. Indeed, it has been proved that lycopene and α-tocopherol together inhibit HL-60 leukemic cell differentiation, low-density lipoprotein (LDL) oxidation, and prostate carcinoma cell proliferation [14]. In the future more detailed studies on the synergistic effects among vitamin E and other phytochemicals in tomato fruit are needed and will open the way for the production of tomato-based functional food.

The amount of tocopherols and tocotrienols in the tomato fruit significantly depends on many biotic and abiotic factors. In particular, the level of irrigation, light, and NaCl exert a high influence on the biosynthesis of these compounds in tomato fruit. However it seems difficult to identify the specific growth conditions to optimize VTE biosynthesis and storage, since many environmental factors combined are responsible for its accumulation. Further investigations are, therefore, desirable in order to know more, in depth, of the impact of each agronomic parameter on VTE accumulation and synthesis. The choice of the superior tomato cultivar is also crucial to obtain tomato fruit enriched with VTE. Today, one main focus of several genetic studies has been the identification of genetic determinants that control the accumulation of VTE in tomato fruit. These studies will help to obtain novel tomato plant lines that accumulate higher levels of VTE in the fruit through conventional breeding programs or, in alternative, through metabolic engineering approaches, thus allowing the introduction of directly-selected key genes into local cultivars. Information coming from the recently completed sequencing of the genome of several tomato varieties and wild relatives can be used to discover new genes and allelic variants. In the future, these might be plausible targets to be engineered in order to improve the VTE content in tomato fruit. The proposed methods might potentially allow obtaining new lines with improved nutritional properties.
